# Model-informed dose optimization for prophylactic piperacillin-tazobactam in perioperative pediatric critically ill patients

**DOI:** 10.1128/aac.01227-24

**Published:** 2025-02-06

**Authors:** Wen Rui Tan, Kei Irie, Carter McIntire, Julie Luna Torres, Rhonda Jones, Abigayle Gibson, Tomoyuki Mizuno, Sonya Tang Girdwood

**Affiliations:** 1 Department of Pharmacology and System Physiology, University of Cincinnati College of Medicine 12303 , Cincinnati, Ohio, USA; 2 Division of Translational and Clinical Pharmacology, Cincinnati Children's Hospital Medical Center 2518 , Cincinnati, Ohio, USA; 3 Hospital Medicine, Cincinnati Children's Hospital Medical Center 2518 , Cincinnati, Ohio, USA; 4 Critical Care Medicine, Cincinnati Children's Hospital Medical Center 2518 , Cincinnati, Ohio, USA; 5 Department of Pediatrics, University of Cincinnati College of Medicine 12303 , Cincinnati, Ohio, USA; University of California, San Francisco, San Francisco, California, USA

**Keywords:** modeling and simulation, population pharmacokinetic, beta-lactam

## Abstract

Piperacillin/tazobactam (PTZ) is frequently prescribed during the perioperative period as prophylaxis in critically ill patients. Current international guidelines recommend that the pediatric intraoperative dosing regimen for PTZ be 90–112.5 mg/kg (80–100 mg/kg as piperacillin [PIP]) administered every 2 hours (Q2H). Concerns have been raised not only about the risk of nephrotoxicity due to elevated PIP exposure but also regarding the practicality of adhering to a 2-h dosing interval in clinical settings. To address these concerns, we employed population pharmacokinetic (PK) modeling and simulation approaches to optimize PTZ dosing regimens in pediatric intraoperative patients. PIP plasma concentration data were obtained from 34 patients using an opportunistic sampling strategy. A two-compartment model was found to adequately describe the PK data. Creatinine clearance was identified as a significant covariate on clearance. The inclusion of inter-occasion variability significantly improved model fit. Simulations across body weights of 10–70 kg and creatinine clearance of 20–130 mL/min/1.73 m^2^ demonstrated that 6–15 mg/kg Q2H, or a 10 mg/kg loading dose followed by 1.0–2.75 mg/kg/h continuous infusion would achieve free PIP concentrations being above the minimum inhibitory concentration (MIC) for 100% of the dosing interval (100% *f*T >1× MIC). For achieving 100% *f*T >4× MIC, 25–55 mg/kg Q2H or a 20 mg/kg loading dose followed by 3.25–9.25 mg/kg/h continuous infusion was derived. The model-informed simulations indicated that both lower Q2H doses and continuous infusion methods are clinically viable options and potentially resolve current clinical challenges during intraoperative dosing.

## INTRODUCTION

There is a significant lack of comprehensive research on pediatric patients undergoing surgical procedures (1). Managing peri-operative medications involves navigating a complex landscape due to the unique physiological responses of each pediatric patient and the critical balance required to manage pain (2), to prevent infection (3), and to facilitate healing. Despite the widespread implementation of antiseptic practices, the risk of postoperative infections remains a significant concern for most surgical procedures (4). This persistent risk underscores the necessity for administering prophylactic antibiotics with precise dosing.

Piperacillin/tazobactam (PTZ), a combination of ureidopenicillin and a beta-lactamase inhibitor, is frequently prescribed for critically ill pediatric patients, including those undergoing abdominal or otolaryngology surgery, due to its broad-spectrum coverage, including anaerobes (5). Additionally, its selection is often justified in preoperative settings due to the high risk of multidrug-resistant organisms or the failure of other antibiotic prophylaxis (6). Achieving optimal plasma concentrations of piperacillin (PIP), the active antibacterial component, is crucial for preventing surgical site infections (SSIs). SSIs continue to be a major contributor to post-surgical morbidity and mortality and are often the primary reason for hospital readmissions following surgery (7).

Current international guidelines recommend that the pediatric intraoperative dosing regimen for PTZ to be 90–112.5 mg/kg (80–100 mg/kg as PIP) administered every 2 hours (Q2H) (6, 8). However, concerns about the potential nephrotoxicity of increased PIP exposure with such high dose highlight the imperative to optimize the PTZ dosing regimen (9). A previous study from our group demonstrated that the average highest PIP C_min_ in the first 24 h of PTZ therapy in patients who developed PIP-associated acute kidney injury (AKI) was 50 mg/L, compared to 10.7 mg/L in those who did not develop PIP-related AKI (9). Additionally, clinical practitioners find the Q2H dosing interval in the operating room to be logistically challenging (personal communication with clinicians who administer antibiotics intraoperatively), suggesting the necessity of revising the current dosing strategy.

The PIP population pharmacokinetic (PK) has been characterized in adult and pediatric critically ill patients (10, 11), while PK data during and after surgery are limited. Surgical procedures can alter physiological parameters such as blood volume, renal function (11), and fluid balance, which in turn can impact PK parameters like clearance and volume of distribution of antimicrobial, such as PIP (12, 13). The alteration in renal function is particularly relevant for PIP, given approximately 68% of the administered dose is excreted unchanged through renal elimination (14). Our previous study suggested considerable inter-individual variability in PIP PK in perioperative pediatric patients (12). This variability underscores the influence of surgical interventions on PIP PK, suggesting the need for individualized dosing strategies for this special population. However, no population PK model for PIP has been established for pediatric patients during and post-surgery.

In this study, we employed model-informed approaches to characterize the PIP population PK and evaluated its optimal dosing regimens to improve pharmacodynamics (PD) target attainment while minimizing the risk of excessive exposure in pediatric patients during surgery.

## MATERIALS AND METHODS

### Patients

We conducted a single-center, open-label opportunistic PK study of PTZ. Data were collected as part of a comprehensive prospective PK investigation of beta-lactam antibiotics in critically ill patients in the pediatric intensive care unit (PICU) at Cincinnati Children’s Hospital Medical Center (CCHMC). The study was approved by the CCHMC Institutional Review Board; a waiver of consent was granted. In the larger prospective study, any patient admitted to the PICU who received at least one dose of PTZ was eligible for enrolment. For the current population PK modeling analysis, we specifically enrolled only those patients who underwent surgery including liver transplantation, Total Pancreatectomy with Islet Auto Transplantation (TPIAT), non-transplant abdominal surgery, and Ear, Nose, and Throat (ENT) surgeries and were treated with high-frequency dosing of PTZ every 2–3 h. For patients below 45 kg, the dosing of PIP was 80–90 mg/kg. For patients above 45 kg, they received the maximum dose of 3,000 or 4,000 mg PIP.

### Sample collection and PIP measurement

A scavenged opportunistic sampling strategy was employed for the purpose of sample collection, whereby residual blood samples were gathered for the measurement of total and free PIP concentrations using a validated high-performance liquid chromatography assay, as described previously (15). Briefly, residual blood from clinical samples drawn after administration of a PTZ dose was requested from the clinical laboratory. This approach enabled the collection of samples intraoperatively and postoperatively for all patients. Additionally, for a subset of patients who were in the PICU before surgery, preoperative samples were also obtained. Samples collected during the 30 min of medication infusion were excluded to allow all the drugs to be infused and distributed. Samples were stored at 4°C for up to 7 days before being centrifuged. Supernatants were stored at −80°C before PIP measurement.

### Population PK modeling

Population PK models were developed for both total and free PIP concentrations. The PK data were analyzed using the Nonlinear Mixed Effect Model (NONMEM) software (version 7.5.1, ICON Development Solutions, Ellicott City, MD, USA) with Perl speaks NONMEM (PsN) version 5.3.0 (16) and Pirana version 21.11.1 (Certara, Princeton, NJ, USA) as the interface, and R version 4.3.3 (R Foundation for Statistical Computing, Vienna, Austria) was used for the PK data exploration, checking for data inconsistencies, graphical evaluation, and model management. While drug efficacy depends on the duration that the free drug concentration remains above the minimum inhibitory concentration (*f*T >MIC), free concentration data are often unavailable in resource-limited settings or necessitate additional processing in routine clinical practice. As a result, the primary analyses were conducted utilizing the total concentration model, while simulations and recommendations for dosing regimens were exclusively based on the free concentration model.

The development of the population PK model was performed stepwise: (i) selection of the structural model; (ii) selection of the error model; (iii) covariate analysis; and (iv) internal validation. One- and two-compartment models with first-order elimination were tested. The parameters were estimated using the first-order conditional estimation method with interaction (FOCE-I). A log-normal distribution was assumed for modeling the inter-individual variabilities (IIV) of the PK parameters. The IIV in PK parameters was evaluated using an exponential error model as follows:


$$\theta_{i}=\theta_{pop}\;\times exp\left(\eta_{i}\right)$$

where $\theta_{i}$ is the population average of the parameter and $\eta_{i}$ is the random effect for individual *i*, the *η* are assumed to be normally distributed with mean 0 and variance *ω*². The inter-occasion variability (IOV) was evaluated as follows:


$$\theta_{i}=\theta_{pop}\;\times \exp\left(\eta_{i}+IOV\right)$$

where IOV is the inter-occasion variability. The IOV is initially set to 0, which corresponds to the intraoperative period. This serves as the baseline or reference point for the model. Then the model estimated separate IOV terms for the pre-surgery and post-surgery periods. A proportional error model was used to describe intra-individual variability. An additive error model and combined residual error model were also tested but did not improve the model fit.

Potential covariates were first selected for testing based on biological plausibility, clinical relevance and later based on the statistical significance. Covariates were tested only on clearance (CL) except for body weight which was also tested on central volume of distribution (*V*1), inter-compartmental clearance (*Q*) and peripheral volume (*V*2). The following covariates were evaluated on CL: body weight (BW), creatinine clearance (CrCL) based on bedside Schwartz equation, postmenstrual age (PMA) for maturation effect, and each surgery type (liver transplantation, TPIAT, non-transplant abdominal surgery, and ENT surgeries). Allometric scaling was used to account for the effect of BW with a power coefficient of 0.75 for CL and *Q*, and 1 for *V*1 and *V*2 as described previously (17). A stepwise covariate analysis was performed utilizing the likelihood ratio test to assess the impact of various factors on the CL of PIP. In forward inclusion, a drop in the objective function value (OFV) of more than 3.84 for 1 degree of freedom was considered statistically significant (*P* < 0.05). After developing a full covariate model, the model reduction was performed through backward elimination, considering an increase in OFV of more than 6.63 for 1 degree of freedom as statistically significant (*P* < 0.01). The following equations were used for the continuous covariate and categorical covariate values:


$$\theta_{i}=\theta_{pop}\;\times \;{\left(\frac{Cov_{i}}{Cov_{median}}\right)}^{\theta_{Cov}}$$

$$\theta_{i}=\theta_{pop}\;\times \theta_{Cov}^{Cov_{i}}$$

where $\theta_{i}$ is the PK parameter estimate for individual *i*, $\theta_{pop}$ is the typical value of the PK parameter in the study population, $\;Cov_{i}$ is the individual value of the covariate, and $\;Cov_{median}$ is the median value of the continuous covariate in the general population. A median value of 70 kg was used for BW and a median value of 120 mL/min/1.73 m^2^ was used for CrCL. $\theta_{Cov}$ is the covariate coefficient. For categorical covariates, $Cov_{i}$ was set to 1 in the presence of the covariate and 0 in its absence.

All models were evaluated by goodness-of-fit (GOF) plots to identify potential bias as a result of model mis-specification. The final model was assessed using a nonparametric bootstrap analysis (*n* = 1,000) and prediction-corrected visual predictive check (pcVPC) (*n* = 1,000) as described previously (18).

### Statistical analysis of patient clinical data and covariates

The normality of the data were assessed using the Shapiro-Wilk test, while the equality of variances was examined using Levene’s test. To determine the statistical significance of *post hoc* analyses of CL in different surgery occasions and surgery types, a Generalized Linear Mixed Model (GLMM) and a parametric one-way analysis of variance (ANOVA) were employed.

### Simulations and dosing design

The model constructed using free PIP concentration data were used to simulate dosing regimens. Two dosing interval regimens (2 hand continuous infusion) were assessed to determine the optimal dosing strategy for the intraoperative period.

The conventional infusion time of 30 min was used for simulation of Q2H dosing regimens. The continuous infusion was simulated by using a 30-min loading dose immediately followed by the continuous infusion (19).

The primary objective of simulation was to achieve and maintain free concentrations above 8 mg/L (100% *f*T >1× MIC), which is the susceptible breakpoint from Clinical and Laboratory Standards Institute (CLSI) for *Enterococcus* and *Enterobacterales* (20), and above 32 mg/L (100% *f*T >4× MIC), throughout the entire surgical procedure. 100% target attainment for >90% of virtual patients was considered adequate. Target attainment was considered to be achieved with the Q2H dosing regimen when the first *C*_trough_ was above the prespecified target attainment; for continuous infusion, target attainment was considered to be achieved when steady-state concentration was above the target concentration during the entire surgery period.

Simulation was performed by using mrgsolve (version 1.4.1) package in R version 4.3.3 (R Foundation for Statistical Computing, Vienna, Austria) (21). A total of 1,000 simulations were conducted for each dosing regimen. In the simulations, both inter-individual variability and inter-occasion variability were accounted for, with an emphasis on accurately simulating the intraoperative phase dose. Patients with different CrCL and BW groups were created to provide tailored doses according to the patient’s renal function and BW. The simulated groups were 20–29, 30–59, 60–89, 90–119, and ≥130 mL/min/1.73 m^2^ (five groups); and 10–29, 30–49, 50–69, and ≥70 kg (four groups), respectively. For simulation purposes, the ≥70 kg weight group was specifically simulated using a range of 70–89 kg, and the ≥130 mL/min/1.73 m^2^ group was simulated using a range of 130–159 mL/min/1.73 m^2^.

## RESULTS

### Patients

Thirty-four patients were included in this study. Demographic characteristics are presented in Table 1. The median (IQR) age and BW were 7.07 years (3.05–14.48) and 20 kg (12.65–55.33), respectively. The median baseline CrCL was 143 mL/min/1.73 m^2^ (102.81–181.53). Twenty-six patients (76%) underwent surgeries involving the abdomen.

**TABLE 1:** Demographic characteristics of patients^*a*^

Characteristic	Value (overall *n* = 34)
Sex (*n*, %)	
Male	15 (44.1%)
Female	19 (55.9%)
Weight (kg)	
Median [IQR]	20.0 [12.65–55.33]
Age (years)	
Median [IQR]	7.07 [3.05–14.48]
Baseline creatinine clearance (mL/min/1.73 m^2^)	
Median [IQR]	143 [102.81–181.53]
Type of surgery (*n*, %)	
Liver transplant	15 (44.1%)
TPIAT	2 (5.9%)
Abdominal	9 (26.5%)
Ear, nose, and throat	8 (23.5%)
Surgery duration (h)	
Median [IQR]	5.32 [2.93–8.89]

^*a*^ Abbreviations: IQR, interquartile range; SD, standard deviation; TPIAT, total pancreatectomy with islet auto transplantation.

### Population PK modeling

A total of 286 total PIP and 285 free PIP concentrations (one sample did not have sufficient volume for free concentration measurement) were available for population PK analyses from 34 patients, with a range of 1–19 data points per patient. The PK parameters were estimated for total and free PIP, separately. Model comparison using Akaike’s information criterion and assessments based on GOF revealed that the two-compartment model provided a superior fit compared to the one-compartment model. This finding was consistent with a previously reported PIP PK model (10). The IIV was estimated for the CL and *V*1. However, due to the sparse sampling nature of the data, there was not enough detailed information to accurately estimate the IIV for the *Q* and *V*2. Consequently, the variability for *Q*2 and *V*2 was assumed to be 0 in the model.

An inclusion of allometrically scaled BW significantly improved model fit (ΔOFV = −36.594). The covariate analysis demonstrated that CrCL is a significant covariate on CL (ΔOFV = −23.332, *P* < 0.05). An inclusion of IOV for preoperative and postoperative stages to distinguish from the intraoperative period significantly improved the model fit (ΔOFV = −14.435, *P* < 0.05). CrCL and BW both positively correlated with PIP CL. The results of other covariate evaluations are summarized in Table S1.

In the final model, CL is represented by the following equation:


$$\theta_{CL}\;\left(L/h\right)=7.1\;\times {\left(\frac{WT}{70}\right)}^{0.75}\times {\left(\frac{CrCL}{120}\right)}^{0.484}$$

### Model evaluation

The final parameter estimates for the total PIP PK model are summarized in Table 2. The GOF plots indicated adequate predictions and no significant misspecifications of the final model (Fig. S1). Notably, there is a small overestimation of concentration at later time points due to the small sample size at those time points (Fig. S1D). The non-parametric bootstrap analysis confirmed the robustness and reliability of the parameter estimates in the PopPK model, as the parameter estimates consistently fell within the bootstrap-derived confidence intervals. (Table 2). The pcVPC demonstrated that simulated concentrations were in reasonable agreement with the observed data (Fig. S2). The parameter estimates and the diagnostic results (i.e., GOF plots, pcVPC, and bootstrap analysis) for the free PIP PK model are summarized in Table S2, Fig. S3 and Fig. S4 all confirming good performance and reliable results.

**TABLE 2:** Parameter estimates for the total piperacillin PK model^*a*^

Parameter	Estimate	RSE (%)	Shrinkage (%)	Bootstrap analysis (*n* = 1,000)		
				Median	95% CI	
					Lower	Upper
CL (L/h/70 kg)	7.1	9	-	6.99	5.97	8.12
*V*1 (L/70 kg)	17.2	16	-	16.6	12.13	20.71
*Q* (L/h/70 kg)	3.25	74	-	3.3	1.54	8.43
*V*2 (L/70 kg)	4.79	43	-	5.1	2.95	9.0
*θ* for CrCL on CL	0.484	30	-	0.44	0.2	0.69
IIV for CL (CV%)	28.7	27	28	28.7	12.2	42.3
IIV for V1 (CV%)	41	21	36	40.8	22.2	59.2
IOV for preoperative (CV%)	37.7	56	78	47.0	23.3	87.2
IOV for postoperative (CV%)	33.5	35	27	34.6	11.5	41.0
Residual variability						
Proportional error (CV%)	37.1	0	7	36.6	32.4	41.0

^*a*^ Abbreviations: CI, confidential interval; CV, coefficient of variation; IIV, interindividual variability; Q, inter-compartmental clearance; RSE, relative standard error; V1, volume of distribution of the central compartment; V2, volume of distribution of the peripheral compartment; -, not applicable.

### Evaluation of surgical impact on clearance using empirical Bayes estimates (EBE)

**Fig 1:**
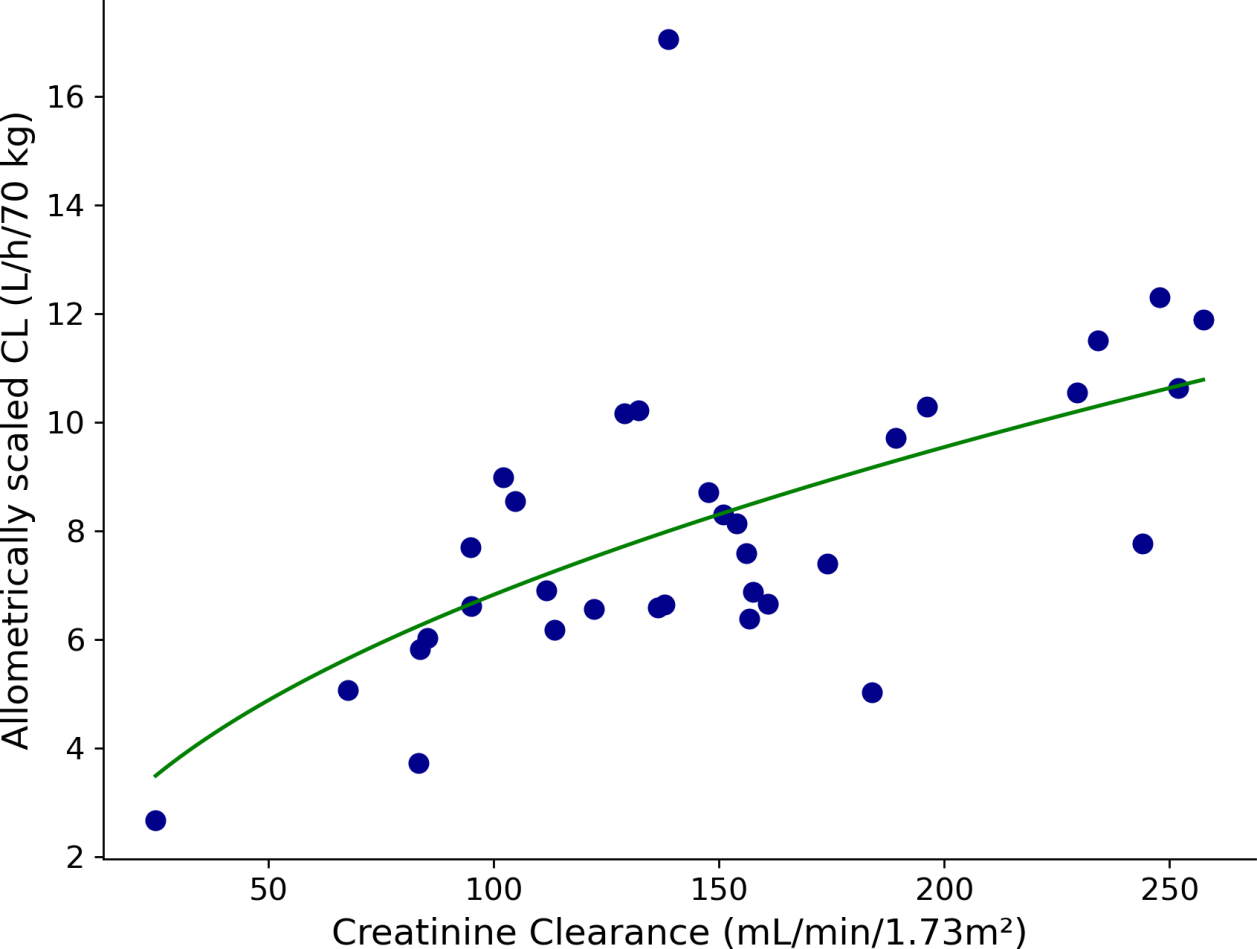
Correlation between baseline CrCL and total piperacillin CL. The green line represents the effect of CrCL on allometrically scaled piperacillin CL [$CLi=CLpop\;\times {\left(CrCL/120\right)}^{0.484}$].

**Fig 2:**
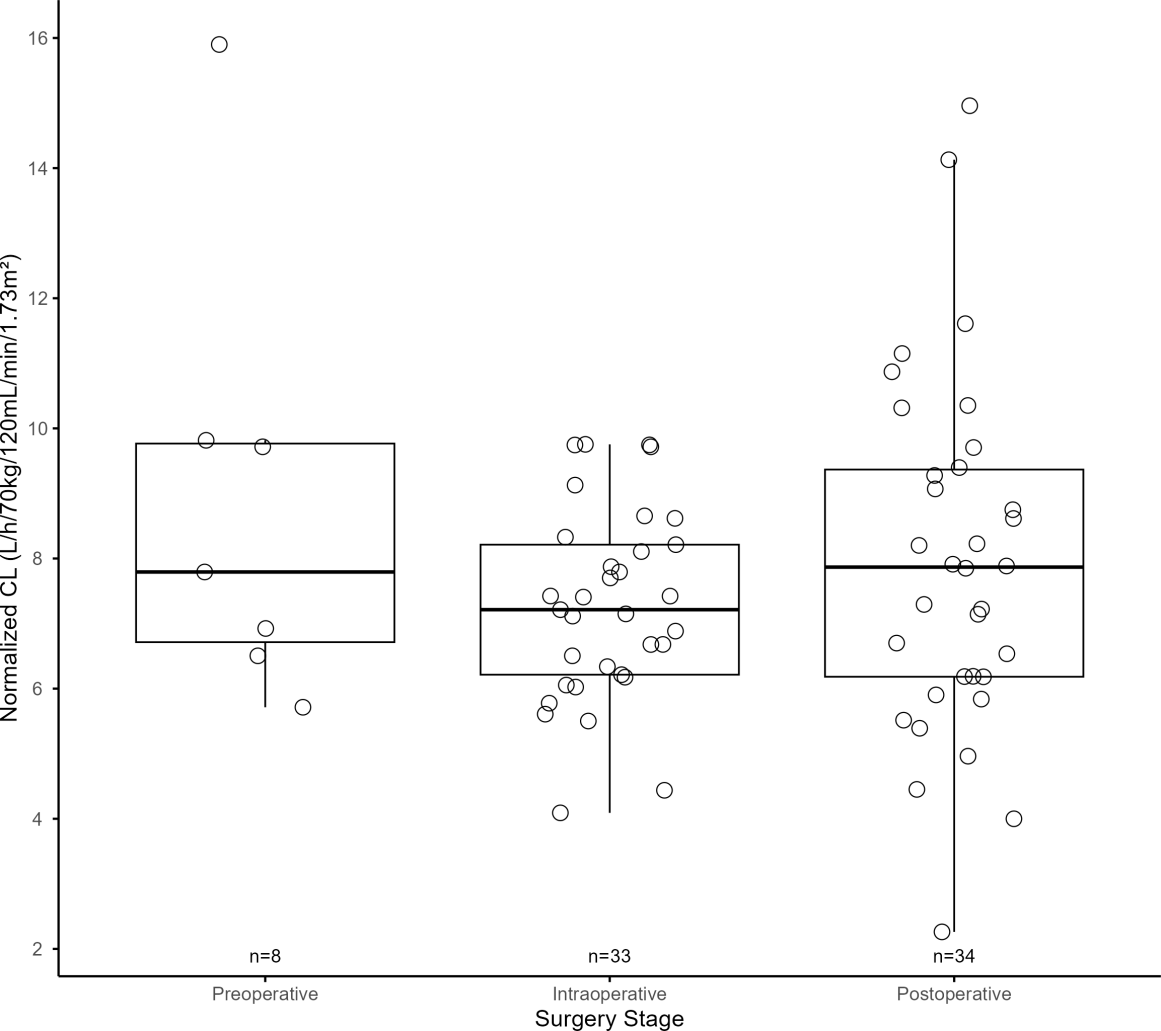
Effect of surgery occasion on CL.

We evaluated the relationship of the covariates on estimated CL *post hoc*. Figure 1 depicts a positive correlation between CrCL and baseline PIP CL, normalized by BW, illustrating their relationship as shown by the estimated clearance equation. Fig. 2 investigates the influence of surgical occasions (preoperative, intraoperative, and postoperative) on CL. This analysis normalized CL relative to BW and CrCL, allowing us to examine the influence of surgical timing on the CL of the drug without confounding factors from the patients’ renal function and BW. The data, subjected to a GLMM analysis, showed non-significant variations in CL across distinct surgical occasions despite a significant IOV based on surgical occasion. Figure S5 and S6 portray the variability in CL observed during and after various surgical interventions, including liver transplant surgery, TPIAT, non-transplant abdominal surgery, and ENT surgeries. Our analysis of the variability of CL across different surgical groups does not encompass preoperative data, as the volume of preoperative data available was insufficient for reliable statistical evaluation after segregating them into different surgical groups. Consequently, our application of the One-way ANOVA test to examine CL focused solely on intraoperative and postoperative periods. This test revealed non-significant results, indicating an absence of significant variations in average CL among the various surgical procedures within our study.

### Simulation and dosing design

**Fig 3:**
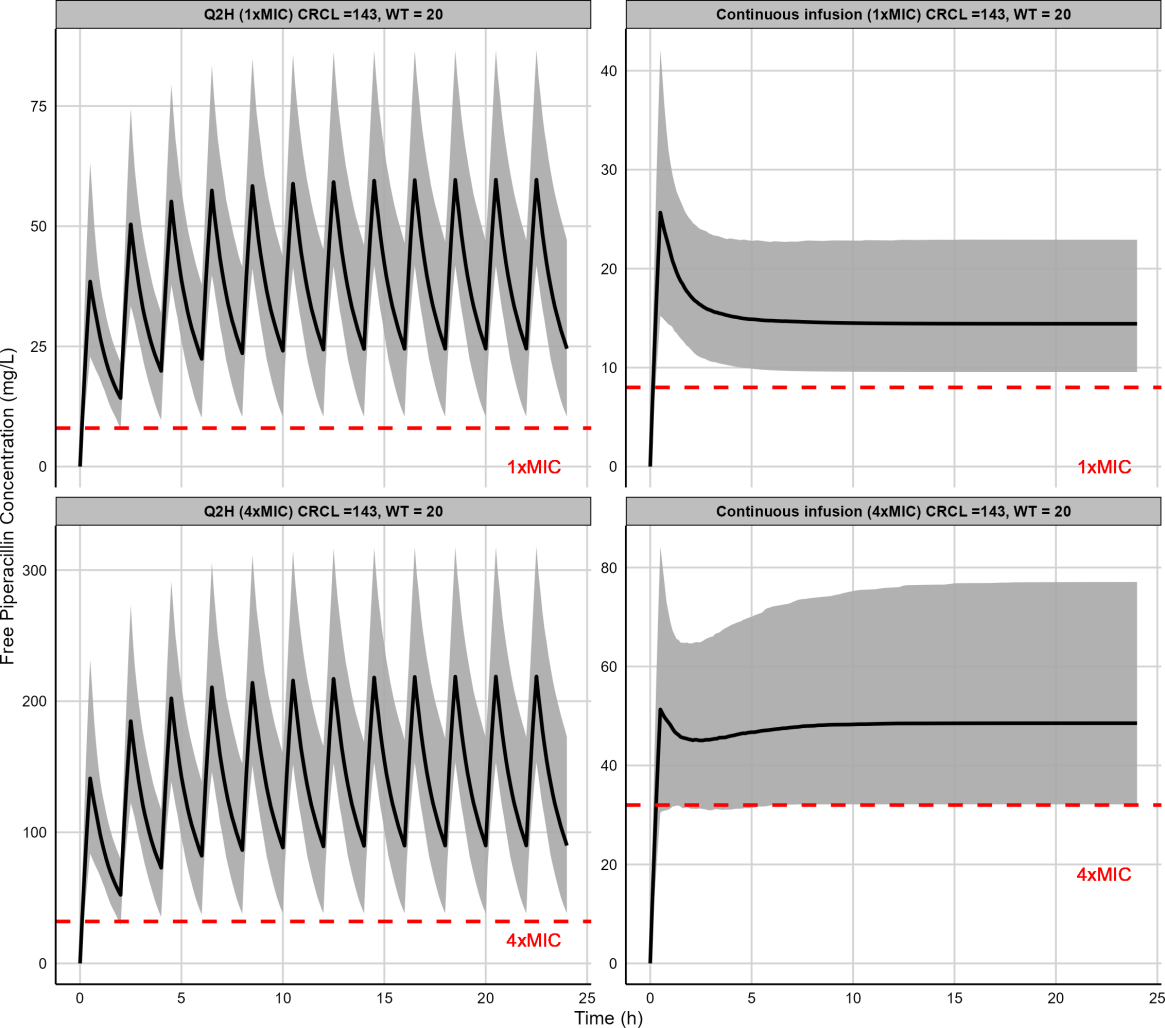
Simulated free PIP concentrations for different dosing regimens in representative virtual patients (*n* = 1,000) with median body weight (20 kg) and median CrCL (143 mL/min/1.73 m^2^) of the current study. The 30-min infusion of 15 mg/kg PIP every 2 hours (Q2H) (top left) and 30-min infusion of 10 mg/kg PIP loading dose followed immediately by 2.75 mg/kg/h (66 mg/kg/day) of PIP (top right) were shown to achieve target of 100%*T* >MIC in >90% of virtual population. The 30-min infusion of 55 mg/kg of PIP Q2H (bottom left) and 30-min infusion of 20 mg/kg PIP loading dose followed immediately by 9.25 mg/kg/h (222 mg/kg/day) of PIP (bottom right) were shown to achieve 100%*T* >4× MIC in >90% of virtual population. The bolded black line indicates the 50th percentile of the simulated concentration. The shaded region represents the 5th and 95th percentiles of the simulated concentration.

Simulations of various dosing regimens for PIP, including Q2H and continuous infusion, were conducted, using an MIC based on the CLSI breakpoint for *Enterococcus* and *Enterobacterales* (8 mg/L). The simulation results for the current dosing regimen, as depicted in Fig. S7 (100 mg/kg PIP Q2H), resulted in excessively high peak and trough concentration of PIP. Figure 3 illustrates that to achieve PK targets of 100% *f*T >MIC and 100% *f*T >4× MIC in 90% of a virtual patient population for the duration of a surgical procedure, dosing regimens can either be 2-h intervals or continuous infusions.

The simulation suggests that the lowest effective PIP dose for 1× MIC for pediatric perioperative patients was found to be 6 mg/kg Q2H (Table 3) for patients with BW ≥70 kg and CrCL of 20–29 mL/min/1.73 m^2^; the highest effective dose for 4× MIC levels reached 55 mg/kg Q2H (Table 4) for patients with BW 10–29 kg and CrCL of ≥130 mL/min/1.73 m^2^, which is significantly lower than current guidelines suggest (80–100 mg/kg Q2H). For continuous infusion, dosing ranged from a loading dose of 10 mg/kg followed by 1.0 to 2.5 mg/kg/h (24–60 mg/kg/day) for 1× MIC and up to 20 mg/kg loading dose followed by a maintenance dose as high as 9.25 mg/kg/h (222 mg/kg/day) for 4× MIC (Tables 3 and 4, respectively). The simulated concentration-time profiles for representative patient populations with different combinations of BW and CrCL are summarized in Fig. S8 and S9.

**TABLE 3:** Dosing recommendations for piperacillin 2 hours interval (Q2H) regimen to achieve 100% *f*T >1× MIC and 100% *f*T >4× MIC, respectively

Regimen and CrCL	Dosing recommendation at weight			
	10–29 kg	30–49 kg	50–69 kg	≥70 kg
Q2H f*T* >1× MIC (8 mg/L)				
20–29	7.5 mg/kg	7 mg/kg	6.5 mg/kg	6 mg/kg
30–59	9 mg/kg	8.5 mg/kg	8 mg/kg	7.5 mg/kg
60–89	9.5 mg/kg	9 mg/kg	8.5 mg/kg	8 mg/kg
90–129	10.5 mg/kg	10 mg/kg	9.5 mg/kg	9 mg/kg
≥130	15 mg/kg	11 mg/kg	10.5 mg/kg	10 mg/kg
Q2H f*T* >4× MIC (32 mg/L)				
20–29	29 mg/kg	27 mg/kg	25 mg/kg	25 mg/kg
30–59	30 mg/kg	30 mg/kg	30 mg/kg	30 mg/kg
60–89	40 mg/kg	38 mg/kg	36 mg/kg	34 mg/kg
90–129	41 mg/kg	39 mg/kg	37 mg/kg	35 mg/kg
≥130	55 mg/kg	45 mg/kg	43 mg/kg	41 mg/kg

**TABLE 4:** Dosing recommendations for piperacillin as a continuous infusion regimen to achieve 100% *f*T >1× MIC and 100% *f*T >4× MIC, respectively

Regimen and CrCL	Dosing recommendation at weight			
	10–29 kg	30–49 kg	50–69 kg	≥70 kg
Continuous infusion (CI) fT >1× MIC (8 mg/L)				
20–29	10 mg/kg LD, 1.25 mg/kg/h CI	10 mg/kg LD, 1.0 mg/kg/h CI	10 mg/kg LD, 1.0 mg/kg/h CI	10 mg/kg LD, 1.0 mg/kg/h CI
30–59	10 mg/kg LD, 2.0 mg/kg/h CI	10 mg/kg LD, 2.0 mg/kg/h CI	10 mg/kg LD, 2.0 mg/kg/h CI	10 mg/kg LD, 2.0 mg/kg/h CI
60–89	10 mg/kg LD, 2.0 mg/kg/h CI	10 mg/kg LD, 2.0 mg/kg/h CI	10 mg/kg LD, 2.0 mg/kg/h CI	10 mg/kg LD, 2.0 mg/kg/h CI
90–129	10 mg/kg LD, 2.5 mg/kg/h CI	10 mg/kg LD, 2.25 mg/kg/h CI	10 mg/kg LD, 2.25 mg/kg/h CI	10 mg/kg LD, 2.0 mg/kg/h CI
≥130	10 mg/kg LD, 2.75 mg/kg/h CI	10 mg/kg LD, 2.5 mg/kg/h CI	10 mg/kg LD, 2.25 mg/kg/h CI	10 mg/kg LD, 2.0 mg/kg/h CI
Continuous infusion f*T* >4× MIC (32 mg/L)				
20–29	20 mg/kg LD, 4.25 mg/kg/h CI	20 mg/kg LD, 4.25 mg/kg/h CI	20 mg/kg LD, 4.25 mg/kg/h CI	20 mg/kg LD, 3.25 mg/kg/h CI
30–59	20 mg/kg LD, 6.0 mg/kg/h CI	20 mg/kg LD, 6.0 mg/kg/h CI	20 mg/kg LD, 6.0 mg/kg/h CI	20 mg/kg LD, 6.0 mg/kg/h CI
60–89	20 mg/kg LD, 7.5 mg/kg/h CI	20 mg/kg LD, 6.0 mg/kg/h CI	20 mg/kg LD, 6.0 mg/kg/h CI	20 mg/kg LD, 6.0 mg/kg/h CI
90–129	20 mg/kg LD, 9.0 mg/kg/h CI	20 mg/kg LD, 7.5 mg/kg/h CI	20 mg/kg LD, 6.0 mg/kg/h CI	20 mg/kg LD, 6.0 mg/kg/h CI
≥130	20 mg/kg LD, 9.25 mg/kg/h CI	20 mg/kg LD, 7.75 mg/kg/h CI	20 mg/kg LD, 7.5 mg/kg/h CI	20 mg/kg LD, 6.5 mg/kg/h CI

## DISCUSSION

This study has characterized the PIP population PK in critically ill pediatric patients who received a prophylactic PTZ during surgery and provided new intraoperative dosing recommendations for pediatric patients using a model-informed simulation approach. To our knowledge, this is the first report on a population PK modeling and model-informed dose optimization study for PTZ in this specific group of pediatric patients. Opportunistic sampling was employed to obtain plasma concentrations, enabling the determination of PK parameters for both total and free PIP. We identified CrCL and BW as significant covariates predictive of PIP CL. Also, including IOV significantly improved model fit. The developed free PIP PK model was employed to determine optimal dosing regimens to achieve the desired target achievements during the surgery phase.

The estimated total PIP CL observed in this study (7.1 L/h/70 kg) was lower than that found in previous studies for non-perioperative critically ill pediatric patients (13.4 and 12.6 L/h/70 kg) (10, 22). Factors associated with the observed lower CL in our population could be due to cases of AKI, often a consequence of surgical stress and hemodynamic instability (23), which can significantly impair renal function and thus reduce the CL of PIP. Furthermore, non-linearity between PTZ dose and PIP CL has been reported at higher doses (51 mg/kg every 6 h) in healthy volunteers and could be even more pronounced at 88.9 mg/kg every 2–3 h in our investigated population (24). This suggests that saturation of renal tubular secretion occurs. Additionally, protein binding saturation may occur, leading to an increased free fraction of PIP, which could contribute to enhanced glomerular filtration. (25). This is further supported by the dosage in the two studies we compared the CL with, which were 75 mg/kg every 6 h, a lower dosage and frequency than in our study.

No significant correlation was found between age and the CL of PIP, after adjusting for BW, despite renal function undergoing maturation in the first 2 years. This lack of age effect may be attributed to the age distribution of the patients included in our study. The population under investigation had a median age of 7 years, with only seven patients (21%) who were below 2 years old. The absence of an age-related impact on PIP CL aligns with our expectations, as developmental changes in organ function, particularly kidney maturation and the ontogeny of drug metabolic enzymes, typically manifest between infancy and early childhood (approximately 0 to 2–3 years of age) (26).

Although the inclusion of IOV significantly improved the model fit, there were no significant differences in individual CL estimates among preoperative, postoperative, and intraoperative (Fig. 2). This may be attributed to a large inter-individual variability in CL and relatively small sample size. Notably, a large IIV was observed in the postoperative period (min–max estimated CL was 2.26–14.96 L/h/70 kg), suggesting the necessity of dose individualization to provide optimal doses for each patient postoperatively. Similarly, there were no significant differences observed in PIP CL among patients who had different types of surgery (Fig. S5 and Fig. S6). Further evaluation with a large sample size is suggested to verify those findings. Dose individualization with plasma concentration monitoring and a model-informed Bayesian adaptive control strategy may provide an attractive approach to optimizing PIP doses for postoperative patients, as successfully utilized in other therapeutic areas (27). A further study is warranted to evaluate such dose individualization strategy, and the developed model can be utilized for model-informed predictions and Bayesian estimations.

In our simulation for the intraoperative dosing, we identified dosing regimens to achieve the targets of 100% *f*T >1× MIC (8 mg/L) and 100% *f*T >4× MIC (32 mg/L) in >90% of the virtual population. Both 30-min infusion with Q2H dosing intervals and continuous infusion dosing regimens achieved this goal. As depicted in Fig. 3, the identified Q2H and continuous infusion dosing strategies effectively mitigate the challenges associated with high peak concentrations predicted for the current dosing regimens recommended by the international guidelines (Fig. S7) (6). Compared to the more traditional dosing regimen requiring 80–100 mg/kg of PIP or 90–112.5 mg/kg PTZ administered Q2H, our recommended Q2H dosing involved a substantially lower quantity of medication (highest effective dose of 15 mg/kg PIP or 55 mg/kg PIP for 100% *f*T >1–4× MIC). This dose reduction is not only cost-effective but also avoids the high peak and potential side effects associated with larger doses. However, for the 100% *f*T >4× MIC target, the Q2H dosing regimens provided a high steady-state C_trough_ (above 50 mg/L, the average maximum free trough concentrations previously shown in patients who developed PIP-associated AKI, in most simulated patients) (9). This suggests the advantages of continuous infusion over the Q2H dosing for patients to achieve higher targets.

This study demonstrates that using the continuous infusion could address the logistical and clinical challenges of administering high-frequency doses and, at the same time, alleviate the high steady-state concentration achieved by Q2H dosing (Fig. S8 and Fig. S9). Notably, a slight overshoot of the loading dose could be observed from the simulated concentration-time profile for continuous infusion to achieve 100% *f*T >1× MIC. The primary reason we chose a loading dose of 10 mg/kg instead of a lower one is that a 10 mg/kg loading dose allows us to achieve the target concentration in 12 min, whereas lowering the loading dose to 5 mg/kg would only allow us to achieve the target in 24 min, which is not ideal considering the purpose of the loading dose is to achieve the target rapidly.

This study has limitations. Due to the limited number of patients younger than 2 years old, the effect of maturation of renal function on PIP clearance was not able to be characterized in this study. Therefore, the dosing regimens identified based on the simulation analyses are only for children older than 2 years. In addition, the potential impact of timing inaccuracies in leftover samples on the variance of certain PK parameters, particularly *Q* and *V*2, was not explicitly accounted for in the analysis and should be considered a limitation. Although the model diagnosis process indicated that the model could predict PIP concentration adequately, a relatively high standard error was observed in the estimation of *Q*. This could be attributed to the sparse sampling nature of opportunistic sampling. Furthermore, our study did not directly assess drug penetration into specific surgical tissues, which may exhibit concentration gradients in less vascularized tissues. However, this concern is less likely to impact the surgeries included in this study—such as liver transplantation, TPIAT, abdominal surgeries, and ENT surgeries—given the vascularized nature of these tissues, which facilitates rapid drug distribution and minimizes potential concentration gradients. In addition, it is why a more stringent target, like 100% *f*T >4× MIC may be considered. To our knowledge, despite the relatively small sample size, this population PK modeling study involves the largest population of critically ill pediatric patients given prophylactic PTZ for potential surgical infections and is considered to provide valuable insights into the clinical implementation of model-informed precision dosing in this special population.

### Conclusion

In this study, we developed a population PK model for PIP in a routine clinical population of pediatric critically ill patients undergoing various surgical procedures. Our findings revealed that CrCL and BW, emerged as significant covariates predictive of PIP CL and inclusion of IOV improves the model performance within this unique patient population. The simulation analyses demonstrated both the 2-h dosing interval and continuous infusion methods are clinically viable options. However, continuous infusion would be preferable as it allows us to achieve and maintain the steady-state concentration at a more ideal level compared to Q2H dosing. These findings contribute to the ongoing endeavors aimed at formulating optimized and personalized dosing regimens for PTZ administration in pediatric surgical patients.

### Study highlights

#### What is the current knowledge on the topic?

The current international guidelines recommend pediatric intraoperative dosing of piperacillin/tazobactam (PTZ) at 90–112.5 mg/kg (80–100 mg/kg as piperacillin [PIP]) every 2 hours (Q2H) for prophylaxis in critically ill patients. Concerns exist about nephrotoxicity risk due to high exposure and the practicality of adhering to the 2-h dosing interval.

#### What question did this study address?

This study aimed to optimize the dosing regimen of PTZ in pediatric patients undergoing surgery, considering the risk of nephrotoxicity and the feasibility of dosing intervals in clinical settings.

#### What does this study add to our knowledge?

By employing a model-informed dose optimization approach, the study proposed alternative dosing regimens of the highest effective dose of 15 mg/kg PIP Q2H and a 10 mg/kg loading dose over 30 min followed by 2.75 mg/kg/h continuous infusion to maintain concentrations above the minimum inhibitory concentration for 100% of the dosing interval (100% *f*T >1× MIC), and 55 mg/kg PIP Q2H and a 20 mg/kg loading dose over 30 min, followed by 9.25 mg/kg/h continuous infusion for 100% *f*T >4× MIC. These regimens ensure target attainment while minimizing the risk of excessive exposure, presenting a significant deviation from current guidelines.

#### How might this change clinical pharmacology or translational science?

The proposed dosing regimen may provide safer and more practical PTZ treatment for pediatric patients undergoing surgery. Further prospective evaluation of established dosing regimens is warranted.

## SUPPLEMENTAL MATERIAL

Supplemental material aac.01227-24-s0001.docx Tables S1 and S2; Figures S1 to S9.

ASM does not own the copyrights to Supplemental Material that may be linked to, or accessed through, an article. The authors have granted ASM a non-exclusive, world-wide license to publish the Supplemental Material files. Please contact the corresponding author directly for reuse.

## References

[1] Grandpierre V, Oltean I, Kaur M, Nasr A. 2022. Addressing barriers to evidence-based medicine in pediatric surgery: an introduction to the Canadian association of paediatric surgeons evidence-based resource. World J Pediatr Surg 5:e000332. doi:10.1136/wjps-2021-000332 36474624 PMC9717324

[2] Chen Q, Chen E, Qian X. 2021. A narrative review on perioperative pain management strategies in enhanced recovery pathways-the past, present and future. J Clin Med 10. doi:10.3390/jcm10122568 PMC8229260 34200695

[3] Habteweld HA, Yimam M, Tsige AW, Wondmkun YT, Endalifer BL, Ayenew KD. 2023. Surgical site infection and antimicrobial prophylaxis prescribing profile, and its determinants among hospitalized patients in Northeast Ethiopia: a hospital based cross-sectional study. Sci Rep 13:14689. doi:10.1038/s41598-023-41834-7 37674035 PMC10482873

[4] Madan M, Mehta G, Weintraub M, Lasagna L, Liang R. 1984. Perioperative prophylaxis with cephalosporins. Clin Pharmacol Ther 36:712–715. doi:10.1038/clpt.1984.246 6499352

[5] Shah PJ, Ryzner KL. 2013. Evaluating the appropriate use of piperacillin/tazobactam in a community health system: a retrospective chart review. P T 38:462–483. 24222978 PMC3814439

[6] Bratzler DW, Dellinger EP, Olsen KM, Perl TM, Auwaerter PG, Bolon MK, Fish DN, Napolitano LM, Sawyer RG, Slain D, Steinberg JP, Weinstein RA, American Society of Health-System Pharmacists (ASHP), Infectious Diseases Society of America (IDSA), Surgical Infection Society (SIS), Society for Healthcare Epidemiology of America (SHEA). 2013. Clinical practice guidelines for antimicrobial prophylaxis in surgery. Surg Infect 14:73–156. doi:10.1089/sur.2013.9999 23461695

[7] Merkow RP, Ju MH, Chung JW, Hall BL, Cohen ME, Williams MV, Tsai TC, Ko CY, Bilimoria KY. 2015. Underlying reasons associated with hospital readmission following surgery in the United States. JAMA 313:483–495. doi:10.1001/jama.2014.18614 25647204

[8] Tornøe CW, Tworzyanski JJ, Imoisili MA, Alexander JJ, Korth-Bradley JM, Gobburu JVS. 2007. Optimising piperacillin/tazobactam dosing in paediatrics. Int J Antimicrob Agents 30:320–324. doi:10.1016/j.ijantimicag.2007.05.014 17631983

[9] Tang Girdwood S, Hasson D, Caldwell JT, Slagle C, Dong S, Fei L, Tang P, Vinks AA, Kaplan J, Goldstein SL. 2023. Relationship between piperacillin concentrations, clinical factors and piperacillin/tazobactam-associated acute kidney injury. J Antimicrob Chemother 78:478–487. doi:10.1093/jac/dkac416 36545869 PMC10169424

[10] De Cock PAJG, van Dijkman SC, de Jaeger A, Willems J, Carlier M, Verstraete AG, Delanghe JR, Robays H, Vande Walle J, Della Pasqua OE, De Paepe P. 2017. Dose optimization of piperacillin/tazobactam in critically ill children. J Antimicrob Chemother 72:2002–2011. doi:10.1093/jac/dkx093 28387840

[11] Alobaid AS, Wallis SC, Jarrett P, Starr T, Stuart J, Lassig-Smith M, Mejia JLO, Roberts MS, Roger C, Udy AA, Lipman J, Roberts JA. 2017. Population pharmacokinetics of piperacillin in nonobese, obese, and morbidly obese critically ill patients. Antimicrob Agents Chemother 61:01276–16. doi:10.1128/AAC.01276-16 PMC5328553 28052849

[12] McIntire C, Luna Torres J, Tang P, Vinks AA, Kaplan J, Tang Girdwood S. 2024. Piperacillin pharmacokinetics and pharmacodynamics in paediatric patients who received high frequency intra-operative piperacillin/tazobactam dosing. Int J Antimicrob Agents 63:107079. doi:10.1016/j.ijantimicag.2023.107079 38161045 PMC10923153

[13] Kennedy JM, Riji AM. 1998. Effects of surgery on the pharmacokinetic parameters of drugs. Clin Pharmacokinet 35:293–312. doi:10.2165/00003088-199835040-00003 9812179

[14] Food and Drug Administration(FDA). 2022. Piperacillin sodium; tazobactam sodium. Available from: https://www.accessdata.fda.gov/scripts/cder/daf/index.cfm?event=overview.process&ApplNo=050684

[15] Tang Girdwood SC, Tang PH, Murphy ME, Chamberlain AR, Benken LA, Jones RL, Stoneman EM, Kaplan JM, Vinks AA. 2021. Demonstrating feasibility of an opportunistic sampling approach for pharmacokinetic studies of β-lactam antibiotics in critically ill children. J Clin Pharmacol 61:565–573. doi:10.1002/jcph.1773 33111331 PMC8061424

[16] Lindbom L, Pihlgren P, Jonsson EN. 2005. PsN-Toolkit--a collection of computer intensive statistical methods for non-linear mixed effect modeling using NONMEM. Comput Methods Programs Biomed 79:241–257. doi:10.1016/j.cmpb.2005.04.005 16023764

[17] Anderson BJ, Holford NHG. 2008. Mechanism-based concepts of size and maturity in pharmacokinetics. Annu Rev Pharmacol Toxicol 48:303–332. doi:10.1146/annurev.pharmtox.48.113006.094708 17914927

[18] Byon W, Smith MK, Chan P, Tortorici MA, Riley S, Dai H, Dong J, Ruiz-Garcia A, Sweeney K, Cronenberger C. 2013. Establishing best practices and guidance in population modeling: an experience with an internal population pharmacokinetic analysis guidance. CPT Pharmacometrics Syst Pharmacol 2:e51. doi:10.1038/psp.2013.26 23836283 PMC6483270

[19] Schoenenberger-Arnaiz JA, Ahmad-Diaz F, Miralbes-Torner M, Aragones-Eroles A, Cano-Marron M, Palomar-Martinez M. 2020. Usefulness of therapeutic drug monitoring of piperacillin and meropenem in routine clinical practice: a prospective cohort study in critically ill patients. Eur J Hosp Pharm 27:e30–e35. doi:10.1136/ejhpharm-2018-001713 32296502 PMC7147564

[20] Manuel C, Maynard R, Abbott A, Adams K, Alby K, Sweeney A, Dien Bard J, Flores II, Rekasius V, Harrington A, Kidd TS, Mathers AJ, Tekle T, Simner PJ, Humphries RM. 2023. Evaluation of piperacillin-tazobactam testing against Enterobacterales by the Phoenix, MicroScan, and Vitek2 tests using updated clinical and laboratory standards institute breakpoints . J Clin Microbiol 61:1617. doi:10.1128/jcm.01617-22 PMC9945575 36719243

[21] Elmokadem A, Riggs MM, Baron KT. 2019. Quantitative systems pharmacology and physiologically-based pharmacokinetic modeling with mrgsolve: a hands-on tutorial. CPT Pharmacometrics Syst Pharmacol 8:883–893. doi:10.1002/psp4.12467 31652028 PMC6930861

[22] Béranger A, Benaboud S, Urien S, Moulin F, Bille E, Lesage F, Zheng Y, Genuini M, Gana I, Renolleau S, Hirt D, Tréluyer J-M, Oualha M. 2019. Piperacillin population pharmacokinetics and dosing regimen optimization in critically ill children with normal and augmented renal clearance. Clin Pharmacokinet 58:223–233. doi:10.1007/s40262-018-0682-1 29862466

[23] Prowle JR, Forni LG, Bell M, Chew MS, Edwards M, Grams ME, Grocott MPW, Liu KD, McIlroy D, Murray PT, et al.. 2021. Postoperative acute kidney injury in adult non-cardiac surgery: joint consensus report of the acute disease quality initiative and perioperative quality initiative. Nat Rev Nephrol 17:605–618. doi:10.1038/s41581-021-00418-2 33976395 PMC8367817

[24] Bulitta JB, Kinzig M, Jakob V, Holzgrabe U, Sörgel F, Holford NHG. 2010. Nonlinear pharmacokinetics of piperacillin in healthy volunteers–implications for optimal dosage regimens. Br J Clin Pharmacol 70:682–693. doi:10.1111/j.1365-2125.2010.03750.x 21039762 PMC2997308

[25] Wallenburg E, Ter Heine R, Schouten JA, Raaijmakers J, Ten Oever J, Kolwijck E, Burger DM, Pickkers P, Frenzel T, Brüggemann RJM. 2022. An integral pharmacokinetic analysis of piperacillin and tazobactam in plasma and urine in critically ill patients. Clin Pharmacokinet 61:907–918. doi:10.1007/s40262-022-01113-6 35377133 PMC9249689

[26] Kearns GL, Abdel-Rahman SM, Alander SW, Blowey DL, Leeder JS, Kauffman RE. 2003. Developmental pharmacology–drug disposition, action, and therapy in infants and children. N Engl J Med 349:1157–1167. doi:10.1056/NEJMra035092 13679531

[27] Mizuno T, Emoto C, Fukuda T, Hammill AM, Adams DM, Vinks AA. 2017. Model-based precision dosing of sirolimus in pediatric patients with vascular anomalies. Eur J Pharm Sci 109S:S124–S131. doi:10.1016/j.ejps.2017.05.037 28526601

