# Supplementary material for: Model-informed dose optimization for prophylactic piperacillin-tazobactam in perioperative pediatric critically ill patients
Source: Antimicrob Agents Chemother. 2025 Feb 6;69(3):e01227-24. doi: 10.1128/aac.01227-24 (PMC11881557; doi:10.1128/aac.01227-24)
Supplement: Supplemental material — Tables S1 and S2; Figures S1 to S9. [file aac.01227-24-s0001.docx]

# Supplementary Files

**Supplementary Table**

**Table S1. Model development and covariate evaluation.**

| **Model** | **OFV (change in OFV from Base Model)** |
| --- | --- |
| Two compartments | 2331.232 (+36.594) |
| Two compartments + Body Weight (Base Model)^1^ | 2294.638 |
| Two compartments + Body Weight + CrCL on CL | 2271.306 (−23.332) |
| Two compartments + Body Weight + IOV on CL | 2280.203 (−14.435) |
| Two compartments + Body Weight + Liver Surgery on CL | 2290.334 (−4.301) |
| Two compartments + Body Weight + TPIAT Surgery on CL | 2293.216 (−1.42) |
| Two compartments + Body Weight + Abd Surgery on CL | 2294.134 (−0.5) |
| Two compartments + Body Weight + ENT Surgery on CL | 2293.374 (−1.264) |
| Two compartments + Body Weight + Age (PMA) on CL | 2294.638 (0.00) |
| Two compartments + Body Weight + CrCL on CL + IOV on CL + Liver Surgery on CL^2^ | 2255.524(−39.114) |
| Two compartments + Body Weight +CrCL on CL + IOV on CL (Final Model) | 2256.359(−38.279) |

^1^Body weight were scaled to 70kg using allometric scaling with 0.75 exponent for CL and Q and linearly scaled for volumes.

^2^Not significant in backward elimination

| **Table S2.** Parameter estimates for the free piperacillin PK model. | | | | | | |
| --- | --- | --- | --- | --- | --- | --- |
|  |  |  |  | **Bootstrap Analysis (n = 1,000)** | | |
|  |  |  |  |  | **95% CI** | |
| **Parameter** | **Estimate** | **RSE (%)** | **Shr. (%)** | **Median** | **Lower** | **Upper** |
| CL (L/h/70 kg) | 8.95 | 11 | - | 8.93 | 7.27 | 10.27 |
| V1 (L/70 kg) | 22.4 | 26 | - | 21.33 | 15.49 | 26.87 |
| Q (L/h/70 kg) | 2.51 | 61 | - | 3.27 | 0.78 | 13.33 |
| V2 (L/70 kg) | 4.48 | 65 | - | 5.18 | 1.87 | 9.39 |
| CrCL for CL | 0.457 | 44 | - | 0.43 | 0.15 | 0.84 |
| IIV for CL(CV%) | 27.1 | 39 | 37 | 27.2 | 10.62 | 42.08 |
| IIV for V1(CV%) | 37.5 | 21 | 39 | 38.77 | 14.97 | 52.75 |
| IOV Preoperative (CV%) | 50.7 | 47 | 76 | 58.05 | 28.33 | 99.80 |
| IOV Postoperative (CV%) | 44.4 | 35 | 21 | 44.99 | 13.60 | 68.16 |
| Residual variability | | | | | | |
| Proportional Error (CV%) | 42.7 | 0 | 6 | 42.27 | 38.52 | 45.92 |
| CI, confidential interval; CV, coefficient of variation; IIV, interindividual variability; RSE, relative standard error; Q, inter-compartmental clearance; V1, volume of distribution of the central compartment; V2, volume of distribution of the peripheral compartment | | | | | | |

**Supplementary figures**

**Figure**


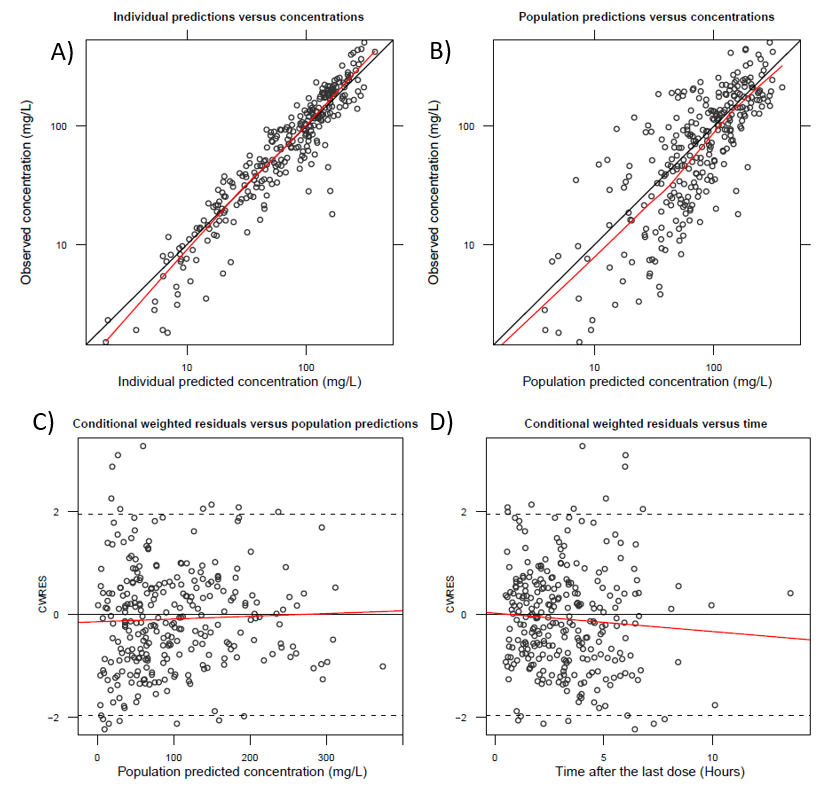
 **Figure S1 Goodness-of-fit plots for the final total piperacillin PK model.** Observed vs. (A) individual-predicted and (B) population-predicted piperacillin concentrations (line of identity shown in red for clarity). The conditional weighted residuals (CWRES) vs. (C) population-predicted piperacillin concentration and (D) time after the last dose


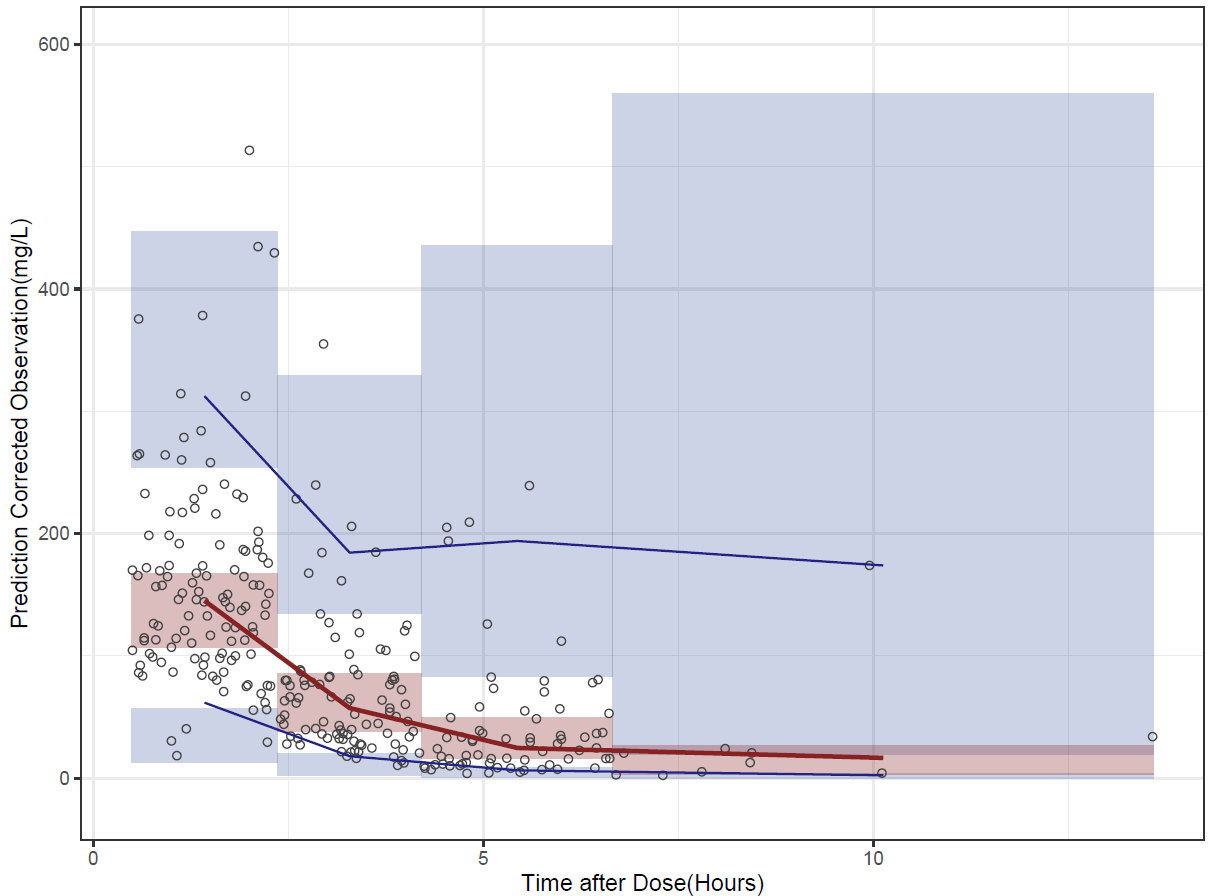


**Figure S2 Prediction-corrected visual predictive check (pcVPC) for the final total piperacillin PK model.** Open circles represent observed plasma concentrations. The red solid line indicates 50^th^ percentile of observation. The blue solid lines indicate 5^th^ and 95^th^ percentile of observations, respectively. The red shaded area represents the 95% confidence interval of 50^th^ percentile of simulation. The blue shaded area represents 95% confidence interval of 5^th^ and 95^th^ percentile of simulation (n = 1,000).


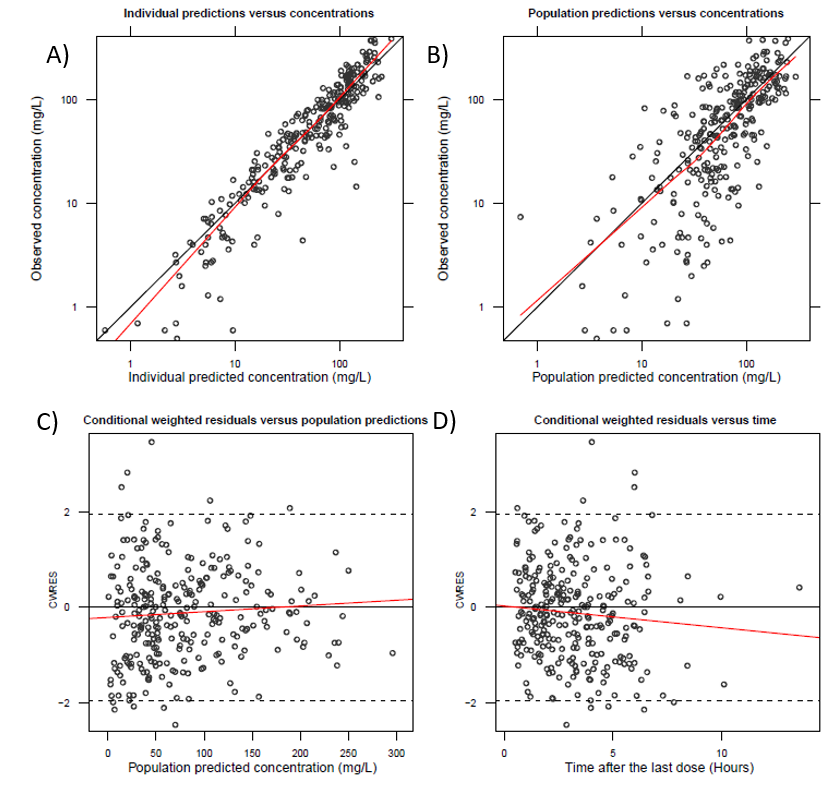


**Figure S3 Goodness-of-fit plots for the final free PIP PK model.** Observed vs. (A) individual-predicted and (B) population-predicted piperacillin concentrations (line of identity shown in red for clarity). The conditional weighted residuals (CWRES) vs. (C) population-predicted piperacillin concentration and (D) time after the last dose

**
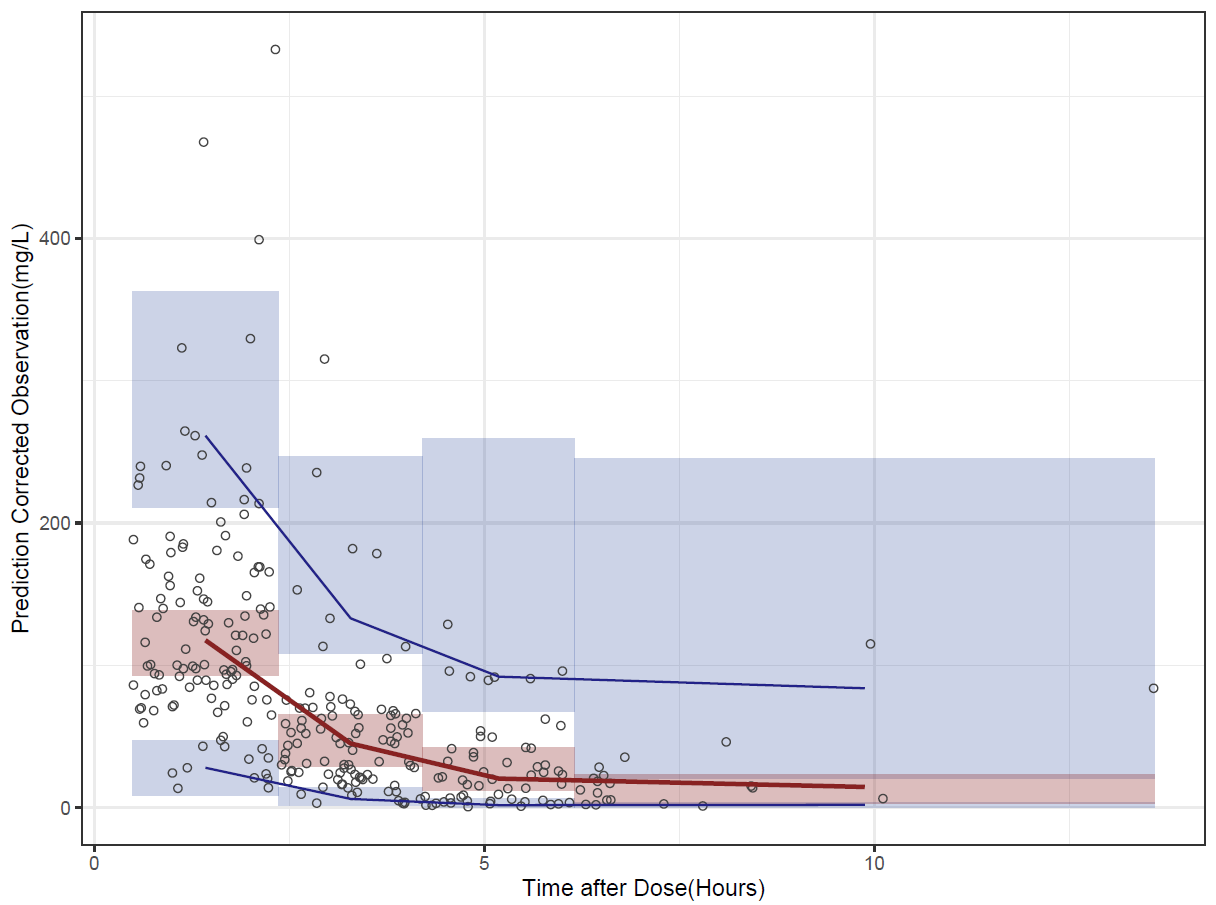
**

**Figure S4 Prediction-corrected visual predictive check (pcVPC) for the final free piperacillin PK model.** Open circles represent observed plasma concentrations. The red solid line indicates 50^th^ percentile of observation. The blue solid lines indicate 5^th^ and 95^th^ percentile of observations, respectively. The red shaded area represents the 95% confidence interval of 50^th^ percentile of simulation. The blue shaded area represents 95% confidence interval of 5^th^ and 95^th^ percentile of simulation (n = 1,000).


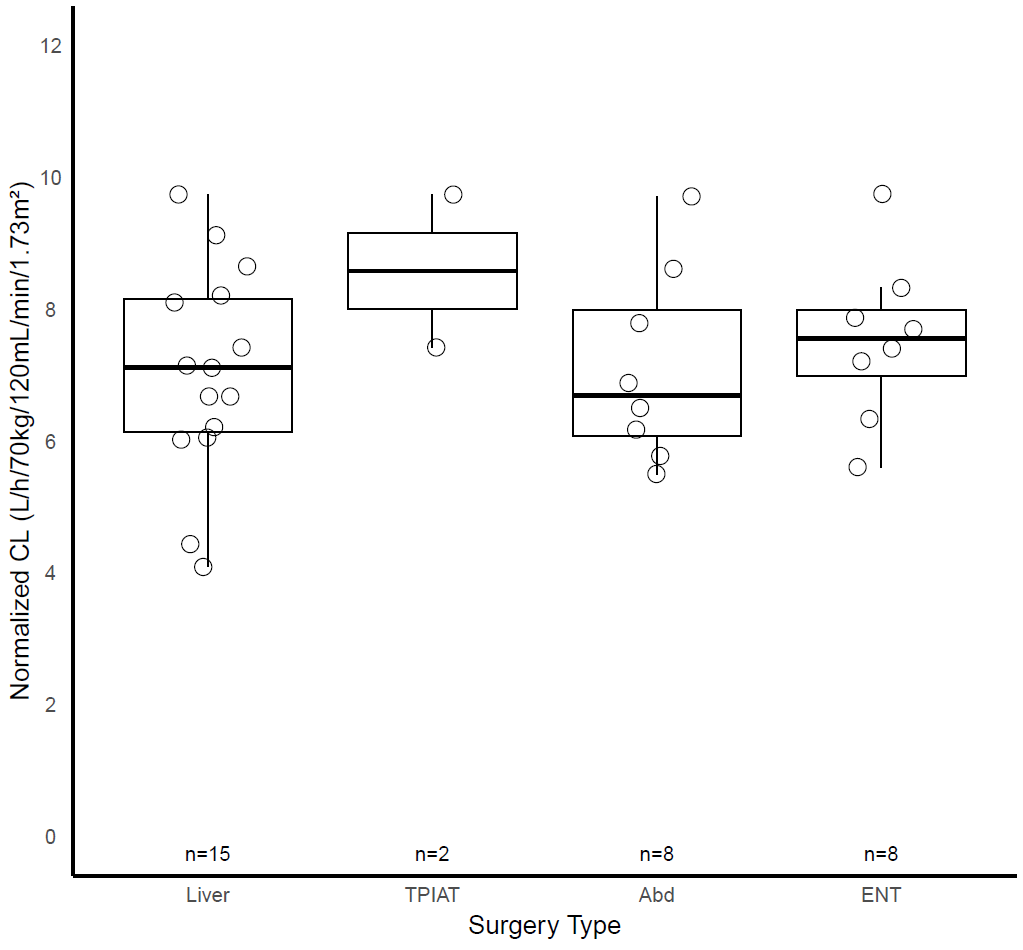


**Figure S5 The effect of type of surgery on total PIP CL during surgery.** Liver transplantation, Liver Transplantation surgery; TPIAT, Total Pancreatectomy with Islet Auto Transplantation; Abdominal surgery, Non-Transplant Abdominal surgery; ENT, Ear, Nose, and Throat surgery


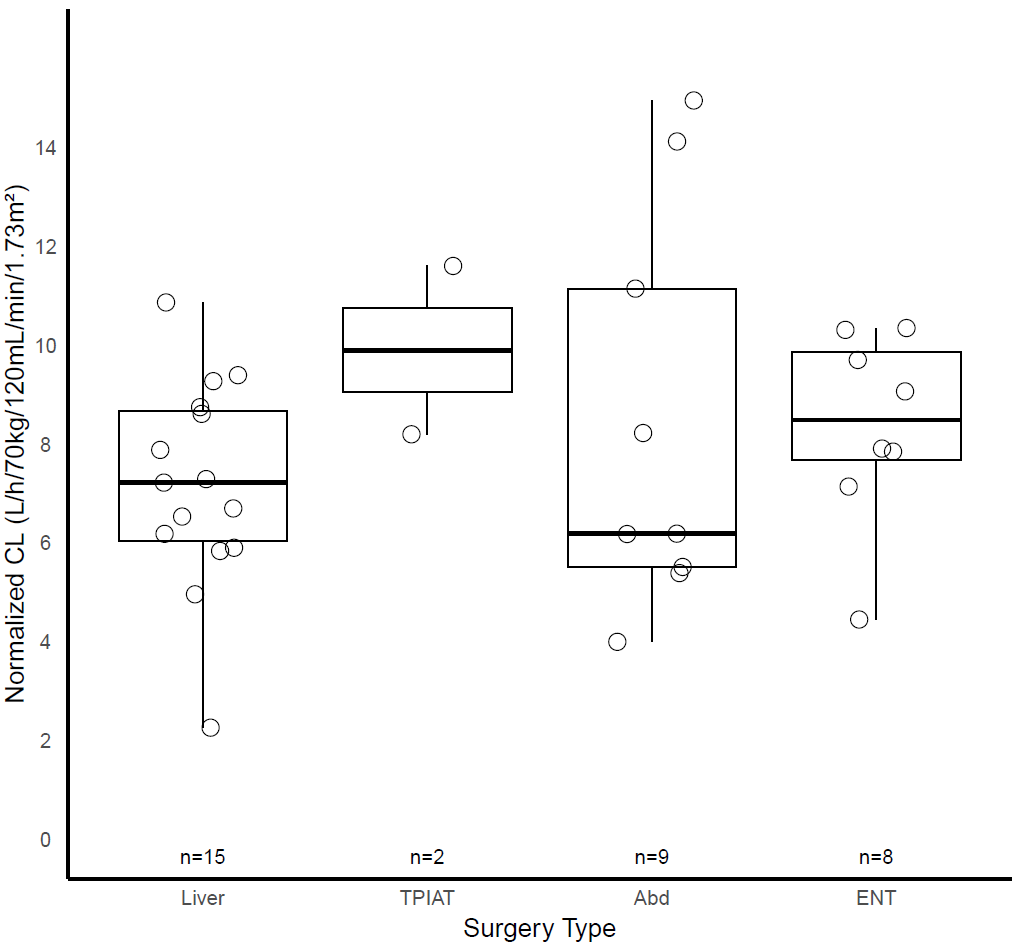


**Figure S6 Clearance Differences between Types of Surgery in Post-Operative Period.** Liver transplantation, Liver Transplantation surgery; TPIRAT, Total Pancreatectomy with Islet Auto Transplantation; Abdominal surgery, Non-Transplant Abdominal surgery; ENT, Ear, Nose, and Throat surgery


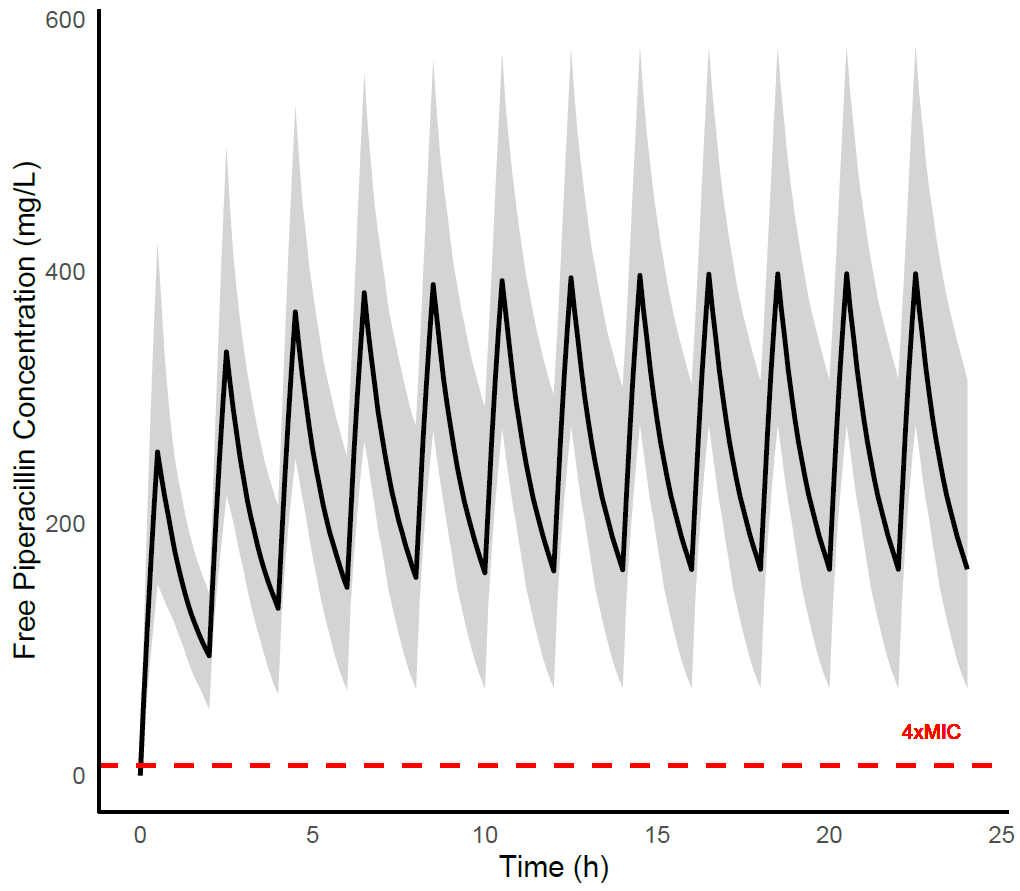


**Figure S7** 100 mg/kg of piperacillin every 2 hours with a 30-minute infusion, the current guideline recommendation (piperacillin/tazobactam 112.5 mg/kg every 2 hours) resulted in excessively high peak and trough concentrations of piperacillin. Median demographic data from this study is used for this simulation. 4xMIC = 32 mg/L.


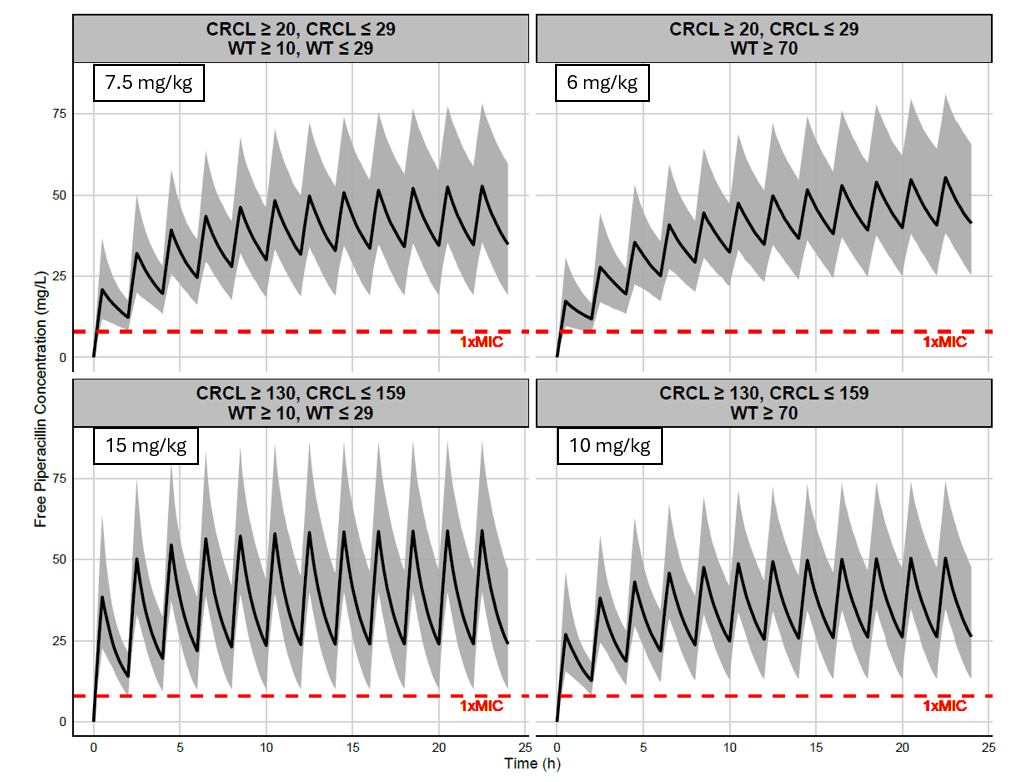


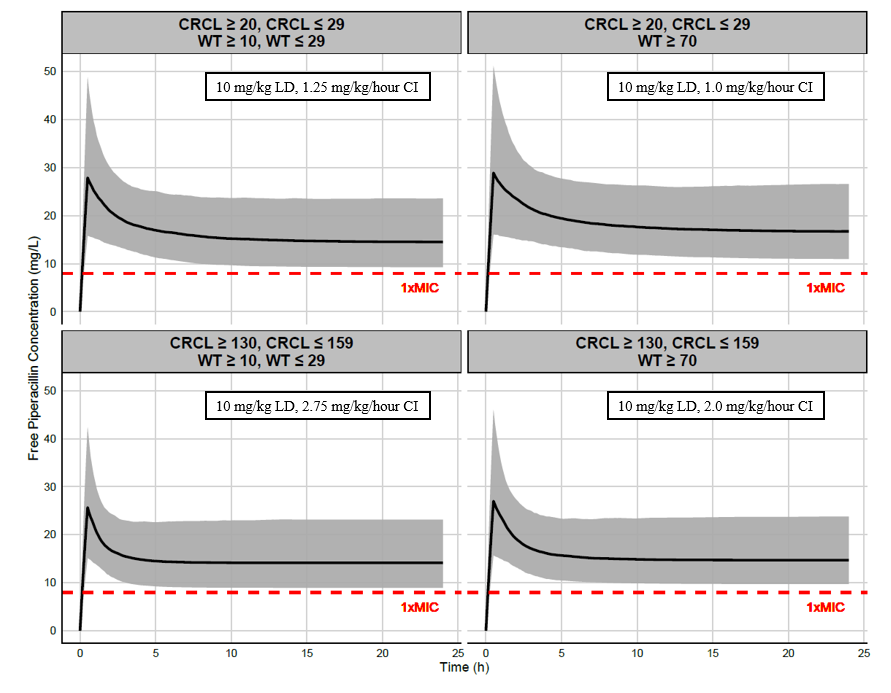


**Figure S8** Simulated free piperacillin concentration-time profile in representative populations for achieving 100% *f*T_>1xMIC_ by Q2H and continuous infusion_._


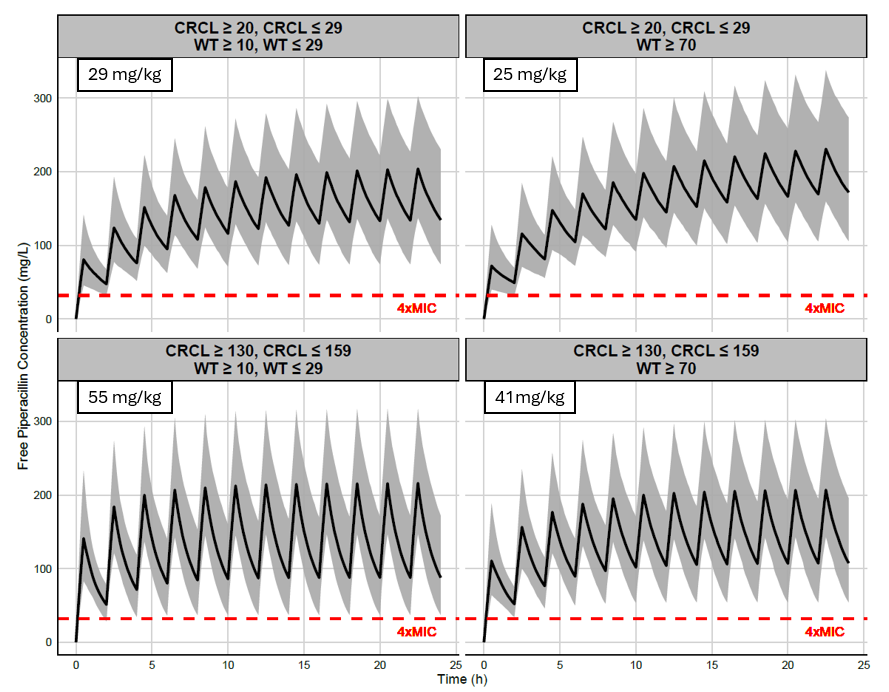


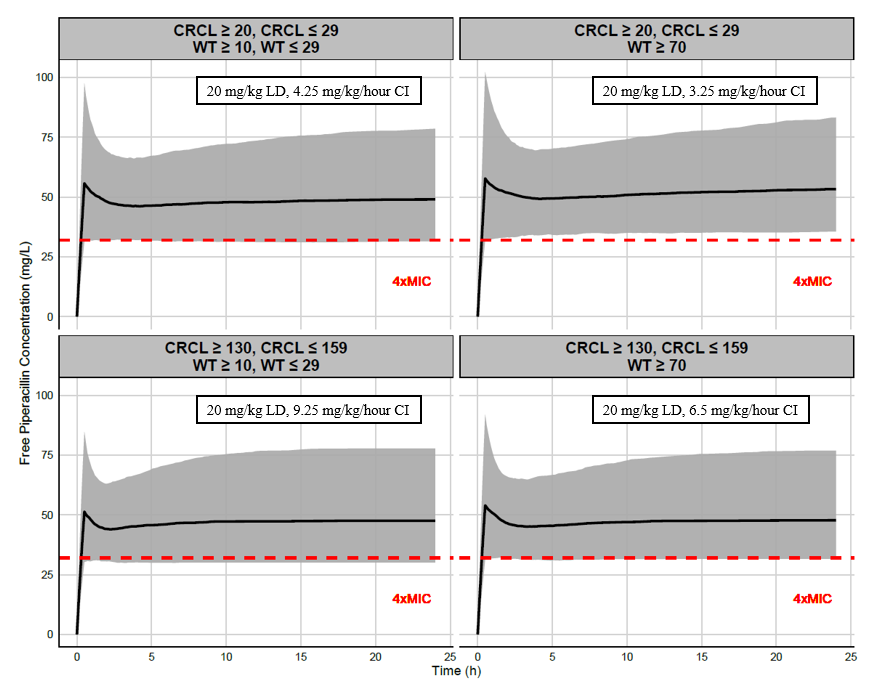


**Figure S9** Simulated free piperacillin concentration-time profile in representative populations for achieving 100% *f*T_>4xMIC_ by Q2H and continuous infusion_._
